# Preparation of Chromatin Fragments From Human Cells for Cryo-EM Analysis

**DOI:** 10.21769/BioProtoc.5472

**Published:** 2025-10-20

**Authors:** Suguru Hatazawa, Yoshimasa Takizawa, Hitoshi Kurumizaka

**Affiliations:** 1 Laboratory of Chromatin Structure and Function, Institute for Quantitative Biosciences, The University of Tokyo, Tokyo, Japan; 2 Department of Computational Biology and Medical Sciences, Graduate School of Frontier Sciences, The University of Tokyo, Tokyo, Japan; 3 Department of Biological Sciences, Graduate School of Science, The University of Tokyo, Tokyo, Japan; 4 RIKEN Center for Integrative Medical Sciences, Yokohama, Japan

**Keywords:** Native chromatin, Native nucleosome, Human cells, Cryo-electron microscopy, Cryo-electron tomography

## Abstract

Eukaryotic genomic DNA is packaged into chromatin, which plays a critical role in regulating gene expression by dynamically modulating its higher-order structure. While in vitro reconstitution approaches have offered valuable insights into chromatin organization, they often fail to fully capture the native structural context found within cells. To overcome this limitation, we present a protocol for isolating native chromatin fragments from human cells for cryo-electron microscopy (cryo-EM) analysis. In this method, chromatin from formaldehyde-crosslinked human HeLa S3 nuclei is digested with micrococcal nuclease (MNase) to generate mono- and poly-nucleosome fragments. These fragments are subsequently fractionated by sucrose-gradient ultracentrifugation and prepared for cryo-EM. The resulting chromatin fragments retain native-like nucleosome–nucleosome interactions, facilitating structural analyses of chromatin organization under near-physiological conditions.

Key features

• Chemical crosslinking preserves the native nucleosome–nucleosome interactions in chromatin fragments.

• Optimal MNase digestion conditions efficiently solubilize chromatin into mono- and poly-nucleosome fragments for cryo-EM analysis.

• This protocol may be adaptable to other types of cells.

## Graphical overview


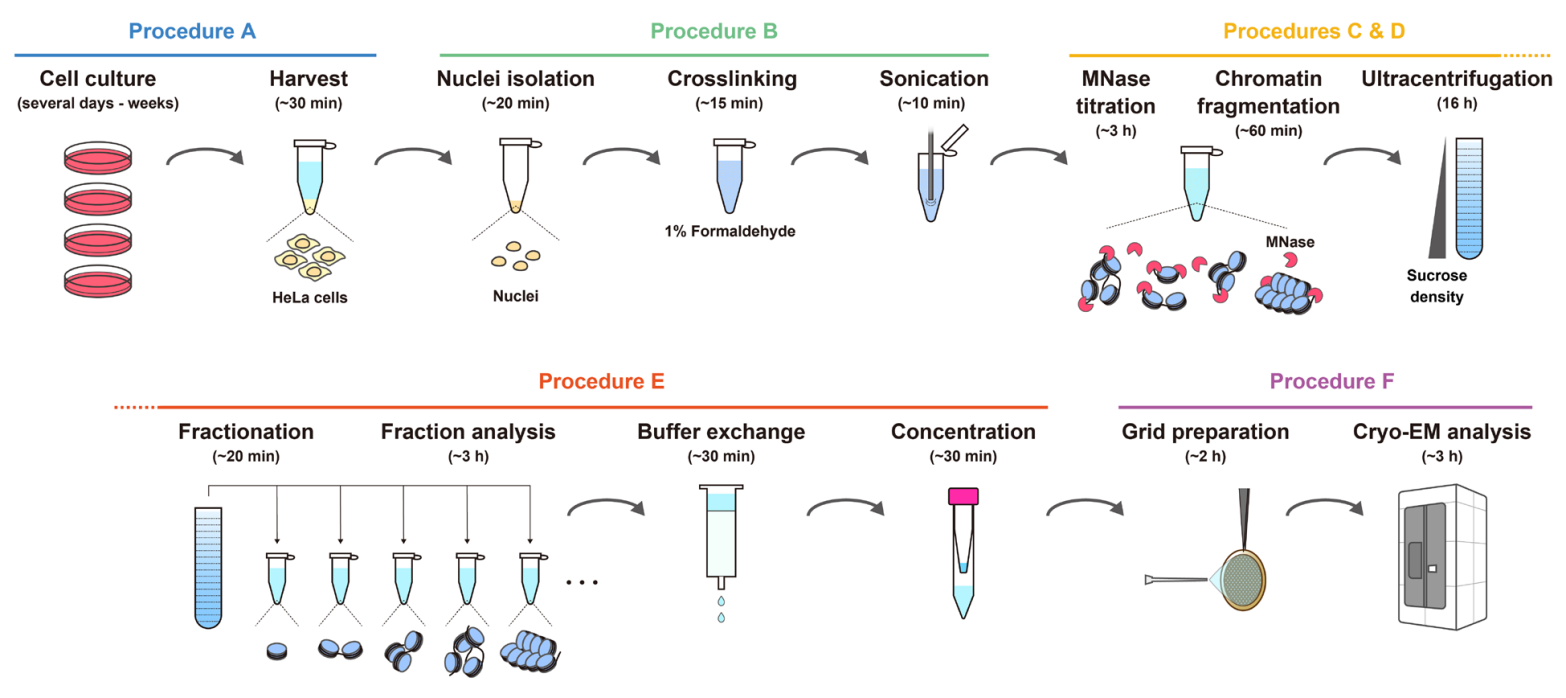


**Workflow for the preparation of chromatin fragments from HeLa cells for cryo-EM analysis.** The estimated times indicate the overall time required for each step, including reactions or incubations.

## Background

Eukaryotic genomic DNA is compactly organized in the nucleus as chromatin, with the nucleosome as its fundamental structural unit [1,2]. The nucleosome consists of approximately 145–147 base pairs of DNA wrapped around a histone octamer, composed of two copies each of histones H2A, H2B, H3, and H4 [3–5]. Structural studies of chromatin have significantly contributed to our understanding of transcriptional regulation, genome stability, and epigenetic inheritance. Biochemical and structural analyses using in vitro–reconstituted chromatin have provided valuable insights into the structural and functional versatility of nucleosomes [6–10]. These reconstituted systems, typically composed of recombinant histones and synthetic DNA templates, offer convenient sample homogeneity and are well-suited for high-resolution structural studies [5,11]. The structures of in vitro–reconstituted nucleosomes are now well characterized, and thus recent efforts have shifted toward understanding their structural and functional properties within the cellular environment, particularly in the context of histone post-translational modifications, native DNA sequences, and inter-nucleosomal interactions.

To better capture chromatin structure within its native cellular context, in situ chromatin analyses using cryo-electron tomography (cryo-ET) have provided significant advancements, aided by sample-thinning techniques such as physical cryosectioning with cryo-microtomes and lamella preparation via cryo-focused ion beam scanning electron microscopy (FIB-SEM) [12–18]. While these approaches are powerful for visualizing chromatin in its native state, they require highly specialized expertise to produce optimal cryo-ET specimens, and access to cryo-FIB-SEM instrumentation remains limited to a small number of research facilities.

To address these limitations, we developed an accessible and robust protocol for preparing native chromatin fragments from HeLa cells for cryo-EM analysis. This method builds on and improves our previously reported chromatin preparation technique [19], combining chemical crosslinking to preserve in situ nucleosome–nucleosome interactions with micrococcal nuclease (MNase) digestion to efficiently solubilize chromatin into mono- and poly-nucleosome fragments. This approach enables high-resolution structural analyses of mono-nucleosomes and provides insights into chromatin organization in a state that closely reflects physiological conditions. Although optimized for HeLa cells, the protocol can be readily adapted to other mammalian cell lines, offering broad applicability for studying chromatin regulation in diverse biological contexts.

## Materials and reagents

**Biological materials**

1. HeLa S3 cells (ATCC, CCL-2.2)

**Reagents**

1. Dulbecco’s modified Eagle medium (DMEM) (Nacalai, catalog number: 08458-16)

2. Fetal bovine serum (FBS) (Gibco, catalog number: 26140-079)

3. Penicillin-streptomycin mixed solution (Nacalai, catalog number: 09367-34)

4. Dulbecco’s phosphate-buffered saline, liquid [D-PBS(-)] without Ca and Mg (Nacalai, catalog number: 14249-95)

5. 2.5 g/L Trypsin/1 mmol/L EDTA solution (Nacalai, catalog number: 35554-64) (used for cell passaging)

6. NE-PER nuclear and cytoplasmic extraction reagents (Thermo Fisher Scientific, catalog number: 78833 or 78835)

7. Protease inhibitor cocktail (EDTA-free) (100×) (Nacalai, catalog number: 03969-34)

8. Formaldehyde solution (36%–38%) (Nacalai, catalog number: 16223-55)

9. Micrococcal nuclease (MNase) (New England Biolabs, catalog number: M0247S)

10. Proteinase K, recombinant, PCR grade (Merck, catalog number: 03115828001)

11. Agarose for 50~800 bp fragment (Nacalai, catalog number: 01147-96)

12. 100 bp DNA ladder (TAKARA, catalog number: 3407A)

13. Ethidium bromide solution (10 mg/mL) (EtBr) (Nacalai, catalog number: 14631-94)

14. Milli-Q water (used directly from the purification system without additional filtration)

15. 2 M glycine (prepared from glycine powder) (Nacalai, catalog number: 17109-35)

16. 1 M Tris-HCl (pH 8.0) (prepared from Tris base) (Nacalai, catalog number: 35434-21)

17. 1 M Tris-HCl (pH 7.5) (prepared from Tris base) (Nacalai, catalog number: 35434-21)

18. 1 M HEPES-NaOH (pH 7.5) (prepared from HEPES) (Nacalai, catalog number: 17514-15)

19. 4 M KCl (prepared from potassium chloride) (Nacalai, catalog number: 28514-75)

20. 5 M NaCl (prepared from sodium chloride) (Nacalai, catalog number: 31320-05)

21. 1 M CaCl_2_ (prepared from calcium chloride) (Nacalai, catalog number: 08894-25)

22. 1 M DTT (prepared from dithiothreitol powder) (Nacalai, catalog number: 14128-62)

23. 0.5 M EDTA (prepared from EDTA 2Na dihydrate) (Nacalai, catalog number: 15130-95)

24. 10% NP-40 (prepared from Nonidet P40 substitute) (Nacalai, catalog number: 18551-24)

25. 30% sucrose (prepared from sucrose powder) (Nacalai, catalog number: 30403-55)

26. 10% SDS (prepared from sodium lauryl sulfate granular) (Nacalai, catalog number: 02873-75)

27. Acetic acid (Nacalai, catalog number: 00212-43)

28. 1× TAE (prepared from Tris base, acetic acid, and EDTA)

**Solutions**

1. Buffer A (see Recipes)

2. Proteinase K mix (see Recipes)

3. 10% sucrose gradient solution (see Recipes)

4. 50% sucrose gradient solution (see Recipes)

5. Buffer B (see Recipes)

**Recipes**

**1. Buffer A**

**Table:** 

Reagent	Final concentration	Quantity or volume
1 M Tris-HCl (pH 8.0)	10 mM	500 μL
4 M KCl	200 mM	2.5 mL
1 M CaCl_2_	1 mM	50 μL
10% NP-40	0.5%	2.5 mL
Milli-Q water	n/a	up to 50 mL
Total	n/a	50 mL

Used for chromatin fragmentation by MNase. Store at 4 °C.

**2. Proteinase K mix**

**Table:** 

Reagent	Final concentration	Quantity or volume
Proteinase K	1/2 of the stock	30 μL
10% SDS (w/v)	1.67%	10 μL
Milli-Q water	n/a	20 μL
Total	n/a	60 μL

Used for digesting proteins in chromatin fragments to estimate DNA size by agarose gel electrophoresis.

**3. 10% sucrose gradient solution**

**Table:** 

Reagent	Final concentration	Quantity or volume
1 M HEPES-NaOH (pH 7.5)	10 mM	500 μL
5 M NaCl	30 mM	300 μL
1 M DTT	1 mM	50 μL
Sucrose	10% (w/v)	5 g
Milli-Q water	n/a	up to 50 mL
Total	n/a	50 mL

Add DTT immediately before use. The lower-density sucrose solution is used for ultracentrifugation.

**4. 50% sucrose gradient solution**

**Table:** 

Reagent	Final concentration	Quantity or volume
1 M HEPES-NaOH (pH 7.5)	10 mM	500 μL
5 M NaCl	30 mM	300 μL
1 M DTT	1 mM	50 μL
Sucrose	50% (w/v)	25 g
Milli-Q water	n/a	up to 50 mL
Total	n/a	50 mL

Add DTT immediately before use. The higher-density sucrose solution is used for ultracentrifugation.

**5. Buffer B**

**Table:** 

Reagent	Final concentration	Quantity or volume
1 M Tris-HCl (pH 7.5)	10 mM	5 mL
5 M NaCl	30 mM	3 mL
1 M DTT	1 mM	500 μL
Milli-Q water	n/a	up to 500 mL
Total	n/a	500 mL

Keep chilled at 4 °C and add DTT immediately before use. Used for buffer exchange after ultracentrifugation prior to cryo-EM sample preparation.

**Laboratory supplies**

1. Dish for tissue culture (for adhesion cell) 100 mm, 300 pieces (IWAKI, catalog number: 3020-100)

2. Cell scraper (Corning, catalog number: 353085)

3. Micropipette tip (Bioland Scientific, catalog numbers: TIPS10, TIPS200, and TIPS1000)

4. Violamo Centrifuge Tube II (AS ONE Corporation, catalog numbers: VIO-15BN and VIO-50BN)

5. Violamo Disposable Pipette II Easy-Open Package (AS ONE Corporation, catalog numbers: 2-5237-11, 2-5237-03, 2-5237-04, 2-5237-05, and 2-5237-06)

6. 1.5 mL hydrophobic microcentrifuge tube, non-sterile, natural, NoStick (Scientific Specialties Inc., catalog number: 1210-10)

7. 13.2 mL Open-Top Thinwall Ultra-Clear Tube, 14 × 89 mm (Beckman Coulter, catalog number: 344059)

8. Prepacked Disposable PD-10 Columns (Cytiva, catalog number: 17085101)

9. Quantifoil R1.2/1.3 200 Mesh, Cu (Quantifoil, catalog number: M2955C-1)

10. C-Clip Ring (Thermo Fisher Scientific, catalog number: 1036173)

11. C-Clip (Thermo Fisher Scientific, catalog number: 1036171)

## Equipment

1. CO_2_ gas incubator (Astec, model: SCA-80DRS)

2. VP-050N ultrasonic homogenizer (TAITEC, model: 0079435-000)

3. Gradient Master (SK BIO International Co., model: 108)

4. Optima XPN-80 (Beckman Coulter, model: A95765)

5. SW 41 Ti Swinging-Bucket Rotor Package (Beckman Coulter, model: 331336)

6. NanoDrop One (Thermo Fisher Scientific, model: ND-ONE-W)

7. PIB-10 (Vacuum Device Inc.)

8. Vitrobot Mark IV (Thermo Fisher Scientific)

9. Krios G4 cryo-TEM (Thermo Fisher Scientific)

10. K3 BioQuantum direct electron detector (Gatan, model: 1967)

## Procedure

The workflow for chromatin fragment preparation from HeLa cells is shown in the Graphical overview.

**A. Cell culture and harvest**

1. Seed HeLa S3 cells in 10 cm culture dishes in 10 mL of DMEM supplemented with 10% FBS and penicillin-streptomycin (1.0 × 10^6^ cells/dish).

2. Culture the cells at 37 °C in a humidified atmosphere containing 5% CO_2_.

3. When the cells reach a subconfluent state (approximately 70%–80% confluency), repeat passaging of the cells.

*Note: A total of 2 × 10^7^ cells (~4 dishes) is the minimum amount required for cryo-EM analysis.*

4. Aspirate the medium and wash the cells twice with 5 mL of PBS prechilled at 4 °C.

5. Harvest the subconfluent cells with a cell scraper.

6. Resuspend the harvested cells in 10 mL of cold PBS and transfer the suspension to a 15 mL conical tube.

7. Count the cells using an automated cell counter or a hemocytometer.

8. Centrifuge at 500× *g* for 5 min at 4 °C and discard the supernatant using a pipette.

9. Place the tube on ice.

**B. Nuclei isolation, crosslinking, and sonication**

1. Resuspend the cell pellet in cold CERI buffer (from NE-PER nuclear and cytoplasmic extraction reagents) to a concentration of 1.0 × 10^7^ cells/mL.

*Note: Nuclei isolation using CERI and CERII buffers was performed entirely according to the manufacturer’s protocol.*

2. Add 1/100 volume of protease inhibitor cocktail.

3. Vortex the tube for 15 s and place it on ice for 10 min.

4. Add 55 μL of cold CERII buffer (from NE-PER nuclear and cytoplasmic extraction reagents) per 1 mL of sample, then vortex for 5 s.

5. Place the tube on ice for 1 min, then vortex for 5 s.

6. Centrifuge at 16,000× *g* for 5 min at 4 °C and discard the supernatant using a pipette.

7. Resuspend the nuclear pellet in cold PBS to a concentration of 1.0 × 10^7^ nuclei/mL, calculated from the initial cell number.

8. Dispense 500 μL of the sample into tubes (0.5 × 10^7^ nuclei/tube).

9. Add formaldehyde to a final concentration of 1% and mix by pipetting and inverting the tube.

10. Incubate the sample for 5 min at room temperature.

11. Add 2 M glycine to a final concentration of 150 mM and mix by pipetting and inverting the tube.

12. Incubate the sample for 3 min at room temperature.

13. Centrifuge at 16,000× *g* for 5 min at 4 °C and discard the supernatant using a pipette.

14. Resuspend the nuclear pellet in buffer A [10 mM Tris-HCl (pH 8.0), 200 mM KCl, 1 mM CaCl_2_, 0.5% NP-40] to a concentration of 2.0 × 10^7^ nuclei/500 μL (4.0 × 10^7^ nuclei/mL) and place it on ice.

*Note: The nuclear pellet does not need to be fully resuspended, as it tends to be very sticky. However, it is advisable to resuspend it as much as possible by pipetting and during the subsequent sonication step (vortexing is ineffective for resuspension).*

**Figure 1.**
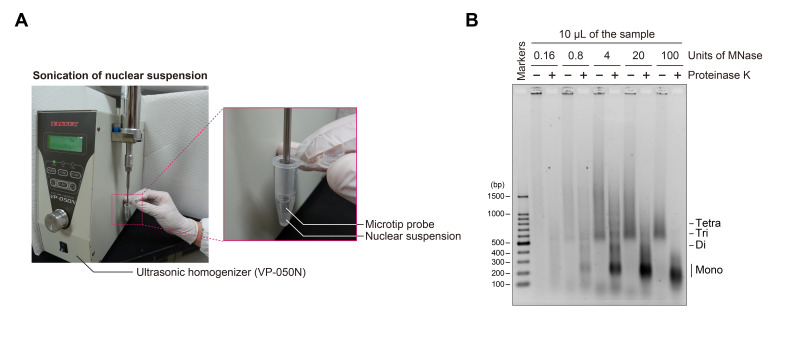
Sonication of nuclear suspension and MNase titration for chromatin fragmentation. (A) Photographs showing the sonication process used for nuclear suspension. (B) Agarose gel electrophoresis of chromatin fragmented by varying units of MNase. A 10 μL portion of each sample with (+) or without (–) proteinase K treatment was applied to the agarose gel, electrophoresed in 1× TAE buffer, and stained with EtBr. The extent of chromatin fragmentation by MNase was estimated based on the nucleosomal DNA bands observed in the proteinase K-treated sample (+).

15. Dispense 500 μL aliquots of the sample into tubes and keep them on ice.

16. Sonicate the nuclear suspension using a VP-050N ultrasonic homogenizer (TAITEC) equipped with a microtip (2 mm in diameter) under the following conditions: PWM = 20%, 3 s on/7 s off, for 6 cycles (Figure 1A).

17. Place the sample on ice.

**C. Titration of MNase for chromatin fragmentation**

1. Prepare MNase solutions at the desired concentrations by performing serial dilutions.

Example: Prepare MNase solutions at 200, 40, 8, 1.6, and 0.32 U/µL.

*Note: Dilute MNase using buffer A, since diluting MNase in Milli-Q water may reduce its enzymatic activity. These MNase concentrations are provided as a reference. Higher concentrations can also be prepared if required.*

2. Collect a portion of the sample and treat it with MNase solution.

Example: Collect 10 μL of the sample into each of five tubes and mix the sample with 0.5 μL of MNase at the indicated concentrations by pipetting.

3. Incubate the samples for 40 min at 37 °C.

4. Add 0.5 M EDTA to a final concentration of 10 mM.

5. Centrifuge at 16,000× *g* for 5 min at 4 °C and collect the supernatants.

6. Place the tubes on ice.

7. Collect a portion of the MNase-treated samples and add the proteinase K mix, then incubate the sample for 15 min at 37 °C.

Example: From the 10 μL MNase-treated sample, 5 μL was mixed with 0.5 μL of the proteinase K mix. The remaining 5 μL was used directly for gel electrophoresis.

*Note: Proteinase K remains active under the 10 mM EDTA conditions used for quenching the MNase digestion.*

8. Add 30% (w/v) sucrose to each sample (with or without proteinase K treatment) to a final concentration of 5% (w/v).

Example: Add 1 μL of 30% sucrose to 5 μL of each sample.

9. Apply the samples to a 1.5% agarose gel and perform electrophoresis in 1× TAE buffer.

10. Soak the gel in EtBr (0.5 µg/mL) and gently shake for 10 min.

11. Transfer the gel to Milli-Q water and gently shake for 10 min.

12. Image the gel using a UV transilluminator (Figure 1B).

*Note: Determine the appropriate MNase concentration for chromatin fragmentation by evaluating the DNA band pattern of proteinase K-treated samples.*

**D. Chromatin fragmentation by MNase and ultracentrifugation**

**Figure 2.**
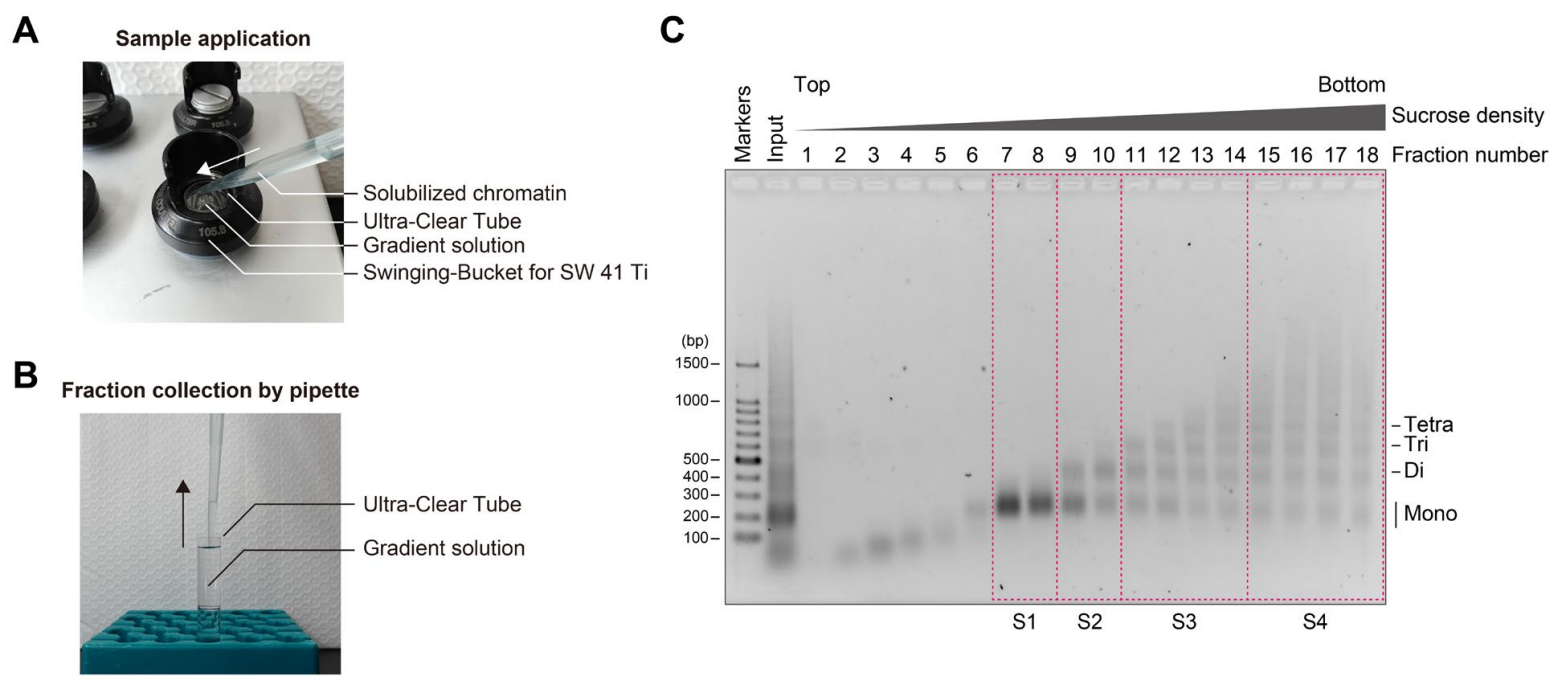
Separation of chromatin fragments by ultracentrifugation. (A) Photograph of sample application onto the top of the gradient solution. (B) Fractionation of chromatin fragments from the gradient solution after ultracentrifugation. (C) Agarose gel electrophoresis of chromatin fractions obtained by sucrose density gradient ultracentrifugation. Chromatin was fragmented with 4 units of MNase per 10 μL of sample and then subjected to ultracentrifugation. A 20 μL portion of each fraction was applied to the agarose gel, electrophoresed in 1× TAE buffer, and stained with EtBr. Fractions enclosed by magenta dashed boxes were combined, subjected to buffer exchange, and subsequently concentrated.

1. Prepare the gradient solution [10%–50% (w/v) sucrose, 10 mM HEPES-NaOH (pH 7.5), 30 mM NaCl, 1 mM DTT] using a Gradient Master 108 at room temperature in ultra-clear tubes (14 × 89 mm).

2. Chill the prepared gradient at 4 °C prior to ultracentrifugation.

3. Pre-incubate the remaining sample for 5 min at 37 °C prior to MNase treatment.

4. Add MNase to the remaining sample, based on the conditions determined in the titration experiment.

5. Incubate the sample for 40 min at 37 °C.

6. Add 0.5 M EDTA to a final concentration of 10 mM.

7. Centrifuge at 16,000× *g* for 5 min at 4 °C and collect the supernatant (~500 μL) using a pipette without disturbing the pellet.

8. Apply the fragmented chromatin onto the top of the gradient solution (Figure 2A).

*Note: As a control for the fractionation step, reserve a portion of the fragmented chromatin sample (as input) prior to centrifugation and store it on ice.*

9. Centrifuge at ~210,000× *g* (35,000 rpm, using an SW 41 Ti rotor) for 21 h at 4 °C, using an Optima XPN-80 ultracentrifuge.

**E. Fractionation, buffer exchange, and concentration of chromatin fragments**

1. Fractionate the solution by collecting 660 μL aliquots from the top of the gradient and place them on ice (Figure 2B).

2. Collect portions of the fractionated samples, add the proteinase K mix, and incubate for 15 min at 37 °C.

Example: Collect 20 μL of the fraction and mix with 2 μL of the proteinase K mix by pipetting. For the input sample, mix 5 μL of the input with 0.5 μL of the proteinase K mix by pipetting.

3. Apply the proteinase K-treated fractions to a 1.5% agarose gel and perform electrophoresis in 1× TAE buffer.

4. Soak the gel in EtBr and gently shake for 10 min.

5. Transfer the gel to Milli-Q water and gently shake for 10 min.

6. Image the gel using a UV transilluminator (Figure 2C).

7. Combine the fractions that contain similar DNA fragment lengths, as estimated from the proteinase K-treated samples.

8. Exchange the buffer of the combined fractions using a PD-10 column (Cytiva) to Buffer B [10 mM Tris-HCl (pH 7.5), 30 mM NaCl, 1 mM DTT].

9. Concentrate the fractions with an Amicon Ultra-4 centrifugal filter unit (30,000 MWCO).

10. Measure the dsDNA concentration of fractions with a NanoDrop One spectrophotometer.

**F. Grid preparation of chromatin fragments**

**Figure 3.**
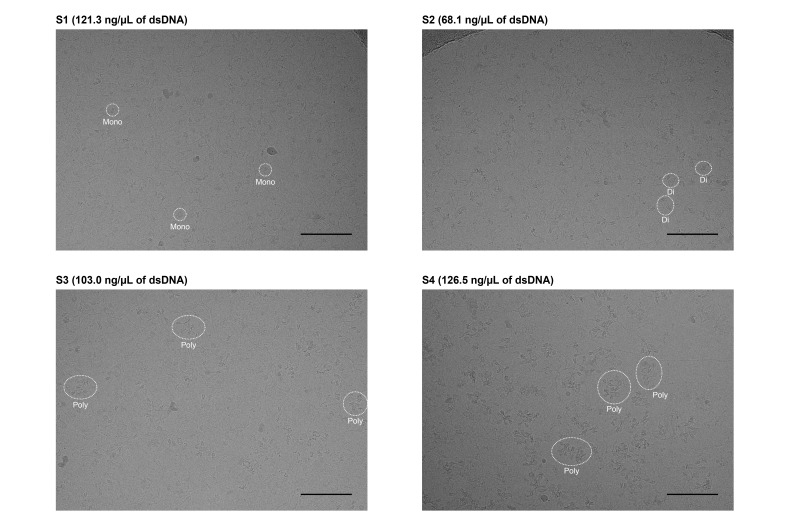
Micrographs of chromatin extracted from HeLa cells. Images were acquired using a Krios G4 cryo-TEM equipped with an energy-filtered K3 detector (Gatan). Data were collected at 300 kV accelerating voltage and 81,000× magnification (corresponding to a pixel size of 1.06 Å), with a defocus setting of -2.5 µm. Each micrograph was generated from 40 movie frames acquired in the electron counting mode over a total exposure time of 4.5 s, resulting in a total dose of ~60 e-/Å^2^. Scale bars represent 100 nm. Representative mono-, di-, and poly-nucleosomes are enclosed by white dotted lines.

1. Hydrophilize Quantifoil R1.2/1.3 200-mesh Cu grids by glow discharge, using a PIB-10 set to soft mode with a 2 min timer.

2. Perform the following steps using a Vitrobot Mark IV at 4 °C under 100% humidity.

3. Apply 2.5 μL of the samples onto the glow-discharged grids.

4. Blot the samples for 4–6 s (wait time: 0 s; blot force: 0; blot total: 1; drain time: 0 s).

5. Plunge the grids into liquid ethane and mount them into a C-clip and C-clip ring.

6. Store the grids in liquid nitrogen.

7. Acquire micrographs using cryo-EM (Figure 3).

## Validation of protocol

This protocol is an optimized version of the chromatin preparation method for cryo-EM analysis previously described in our original research article:

- Hatazawa et al. [19]. Cryo‐EM structures of native chromatin units from human cells. *Genes to Cells* (Figures 1–4 and Figure S1).

## General notes and troubleshooting

**General notes**

1. This protocol was optimized for HeLa S3 cells. Application to other mammalian cell lines may require adjustments of several parameters, such as the concentration of formaldehyde and the incubation time for crosslinking, the sonication output and cycle number, and the MNase units, reaction time, and incubation temperature used in the digestion for chromatin fragmentation.

2. Formaldehyde should be freshly prepared or opened immediately before use to ensure optimal crosslinking efficiency.

**Troubleshooting**

Problem 1: Chromatin is not efficiently solubilized.

Possible cause: Insufficient MNase activity or suboptimal concentration; incomplete resuspension of purified nuclei.

Solution: Prepare MNase dilutions in Buffer A immediately before use to preserve enzymatic activity. Perform titration experiments to determine the optimal MNase concentration for efficient chromatin digestion. Ensure thorough resuspension of nuclei prior to MNase treatment and optimize sonication parameters as necessary to enhance chromatin fragmentation.

Problem 2: Nucleosomes are not well separated by size after ultracentrifugation.

Possible cause: The sucrose gradient does not match the target nucleosome fragment size range, or the gradient was inconsistently prepared.

Solution: Optimize the sucrose concentration range and gradient slope according to the expected size distribution of nucleosome fragments. Prepare the gradient using a Gradient Master 108 at room temperature, then chill the gradient at 4 °C prior to ultracentrifugation. When applying the sample, carefully place it onto the top of the gradient to avoid disturbing the interface.
